# Computer-Aided Design of α-L-Rhamnosidase to Increase the Synthesis Efficiency of Icariside I

**DOI:** 10.3389/fbioe.2022.926829

**Published:** 2022-06-21

**Authors:** Jia-Jun Huang, Hao-Xuan Hu, Yu-Jing Lu, Ya-Dan Bao, Jin-Lin Zhou, Mingtao Huang

**Affiliations:** ^1^ School of Food Science and Engineering, South China University of Technology, Guangzhou, China; ^2^ Golden Health Biotechnology Co., Ltd., Foshan, China; ^3^ School of Chemical Engineering and Light Industry, School of Biomedical and Pharmaceutical Sciences, Guangdong University of Technology, Guangzhou, China

**Keywords:** icariside Ⅰ, icaritin, homology modeling, molecular docking, α-L-rhamnosidase

## Abstract

Icariside I, the glycosylation product of icaritin, is a novel effective anti-cancer agent with immunological anti-tumor activity. However, very limited natural icariside I content hinders its direct extraction from plants. Therefore, we employed a computer-aided protein design strategy to improve the catalytic efficiency and substrate specificity of the α-L-rhamnosidase from *Thermotoga petrophila* DSM 13995, to provide a highly-efficient preparation method. Several beneficial mutants were obtained by expanding the active cavity. The catalytic efficiencies of all mutants were improved 16–200-fold compared with the wild-type TpeRha. The double-point mutant DH was the best mutant and showed the highest catalytic efficiency (*k*_*cat*_/*K*_*M*_: 193.52 s^−1^ M^−1^) against icariin, which was a 209.76-fold increase compared with the wild-type TpeRha. Besides, the single-point mutant H570A showed higher substrate specificity than that of the wild-type TpeRha in hydrolysis of different substrates. This study provides enzyme design strategies and principles for the hydrolysis of rhamnosyl natural products.

## Introduction

Icariside I and icaritin are the main active compounds of herbal medicinal plants *Epimedium* spp. (Li et al., 1996). Icaritin (Zhu and Lou, 2005; Huang et al., 2007; Li et al., 2011) was recently approved for advanced hepatocellular carcinoma as an icaritin soft capsule. Icariside I has effective immunological anti-tumor activity (Chen et al., 2021) and may have better potential than icaritin in the treatment of certain diseases through reversion of tumor immune desertification. Both icariside I and icaritin belong to the *Epimedium* flavonoids (Ma et al., 2011), which consist of primary flavonoid glycosides, secondary flavonoid glycosides, and the aglycone. Primary flavonoid glycosides include epimedin A, epimedin B, epimedin C, icariin, etc. Sagittatoside A, sagittatoside B, icariside I, baohuoside I (icariside II), and icaritin are secondary flavonoid glycosides and the aglycone (Xu et al., 2013).

Although direct extraction is a way to obtain active chemicals from plants, the extremely low content of icaritin and icariside I in *Epimedium* spp. (lower than 0.1%) limits its large-scale preparation. Due to the complex processes, low efficiency, and environmental unfriendliness of icaritin chemical synthesis (Mu et al., 2013), preparation of icariside I and icaritin by the chemical method is greatly limited yet. Therefore, alternative methods are necessary to meet the huge demand for icariside I and icaritin. With the rapid development of biotechnology and synthetic biology, biosynthesis of icariside I and icaritin has become feasible. Recently, Wang et al. (2021) achieved *de novo* biosynthesis of icaritin. When co-culturing the 8-prenylkaempferol-producing yeast with an *Escherichia coli* strain expressing GmOMT2, the yield of icaritin reached 19.7 mg/L. Besides *de novo* synthesis, the biosynthesis of icaritin can also be carried out through a “reverse synthesis” by hydrolyzing glycosyls from epimedin C or icariin (Xie et al., 2020; Cheng et al., 2022). It was reported that both epimedin C and icariin have a rich source in the *Epimedium* spp.; epimedin C accounts for 20.8% of the total *Epimedium* flavonoids, while icariin accounts for 21.9% (Ye et al., 2007; Ma et al., 2011). It is only neccessary to use a glycoside hydrolase for a multi-step hydrolysis to generate icariside I and icaritin from epimedin C or icariin.

Glycoside hydrolases (EC 3.2.1) are a class of enzymes that hydrolyze glycosidic bonds, and they play an important role in hydrolysis and synthesis of sugars and glycoconjugates in organisms (Manzanares et al., 2007). Icariside I can be produced from epimedin C by a two-step rhamnosyl hydrolysis, or produced from icariin by a one-step rhamnosyl hydrolysis. α-L-rhamnosidase is the enzyme widely used in industry to hydrolyze rhamnosyl-based natural products, e.g., rutin, hesperidin, and naringin. Only a few studies have reported the hydrolysis of epimedin C by α-L-rhamnosidases. Moreover, most of those reported α-L-rhamnosidases, such as AnRhaE from *Aspergillus nidulans* (Lyu et al., 2019) and BtRha from *Bacteroides thetaiotaomicron* VPI-5482 (Wu et al., 2018), could only hydrolyze the outer rhamnosidic bond. The inner rhamnosidic bond of epimedin C and icariin could only be hydrolyzed by three α-L-rhamnosidases up to now, TpeRha from *T. petrophila* DSM 13995 (Xie et al., 2020), DthRha from *Dicyolomus thermophilum* DSM 3960 (Zhang et al., 2021), and Rhase-Ⅰ from *Talaromyces stollii* CLY-6 (Cheng et al., 2022). However, the catalytic efficiency of TpeRha and DthRha is insufficient, while the thermostability of Rhase-Ⅰ is poor. The weaknesses of these wild-type enzymes restrict their use in large-scale production. Improvement of these enzymes is required for industrial application in preparation of Icariside I.

Protein engineering, in the forms of directed evolution, rational protein design, computer-aided protein design (Khan et al., 2016), and machine-learning-based protein design (Saito et al., 2018; Yang et al., 2019), is currently the most powerful method for optimizing enzyme properties. Improved performances of enzymes include in catalytic efficiency (Li et al., 2019), thermal stability (Huang et al., 2021), substrate selectivity (Li et al., 2020), pH tolerance, etc. For instance, an α-L-rhamnosidase mutant with improved thermostability obtained by directed evolution and site-directed mutagenesis showed higher efficiency in hydrolyzing naringin for debittering orange juice (Li et al., 2018). The reverse hydrolysis capacity of α-L-rhamnosidase was enhanced in catalytic activity and widely pH tolerance through site-directed mutagenesis in the enzymatic active pocket (Xu et al., 2016). A double-site mutant α-L-rhamnosidase with higher thermostability was obtained by site-directed mutagenesis based on homology modeling, molecular docking, and molecular dynamic studies (Liao et al., 2019).

In this study, several α-L-rhamnosidase mutants were successfully obtained by computer-aided protein design techniques, including homology modeling and molecular docking. The design rationale was based on adjusting the steric influence of amino acids around the ligand-binding pocket (LBP) of the enzyme. These mutants showed higher hydrolysis efficiency and greater substrate specificity in conversion from icariin to icariside I. Our findings provide design principles and techniques for building α-L-rhamnosidase mutants with high catalytic efficiency and high substrate selectivity. α-L-rhamnosidase mutants identified here also pave the way for the industrial production of icariside I.

## Materials and Methods

### Strains, Reagents, and Chemicals

The α-L-rhamnosidase gene of *T. petrophila* DSM 13995 (*TpeRha*, GenBank accession: ABQ47687.1) was codon-optimized, synthesized and cloned into the vector pET-28a by General Biosystems Co., Ltd. (Anhui, China). Details of strains and plasmids used in this study are listed in Table 1. Primers are listed in the Supplementary Table S1. Competent cells of *E. coli* BL31(DE3) and *E. coli* DH5α were purchased from Weidi Biotechnology Co., Ltd. (Shanghai, China). Plasmid Mini Kit I was purchased from Omega Bio-tek Inc. (Georgia, United States). The PrimeSTAR^®^ Max DNA Polymerase, used for site-directed mutagenesis, was purchased from Takara Biomedical Technology Co., Ltd. (Dalian, China). Restriction enzymes were purchased from Thermo Fisher Scientific Co., Ltd. (Beijing, China). The HiPure Gel Pure DNA Micro Kit was acquired from Magen Co., Ltd. (Guangzhou, China). The standards epimedin C, naringin, rutin, hesperidin, naringin dihydrochalcone (NDHC), icariin, and icariside I were purchased from Shanghai yuanye Bio-Technology Co., Ltd. (Shanghai, China). The epimedin C and icariin was purchased from Tianben Bio-Engineering Co., Ltd. (Xian, China). Other general chemical reagents used in this study were obtained from standard suppliers.

**TABLE 1:** Strains and plasmids used in this study.

Strain/plasmid	Description	Source or reference
Strains		
*Escherichia coli*		
DH5α	F^−^ φ80 *lac* ZΔM15 Δ(*lac*ZYA-*arg* F) U169 *end*A1 *rec*A1 *hsd*R17(r_k_^−^,m_k_^+^) *sup*E44λ- *thi* -1 gyrA96 *rel*A1 *pho*A	Weidi Biotechnology Co., Ltd. (Shanghai, China)
BL21 (DE3)	F^−^ *omp*T *hsd*S_B_(r_B_^−^ m_B_^−^) *gal dcm*(DE3)	Weidi Biotechnology Co., Ltd. (Shanghai, China)
BL21 (DE3)/pET-28a-TpeRha	BL21(DE3) engineered strain of wild-type TpeRha	This study
BL21 (DE3)/pET-28a-E462A	BL21(DE3) engineered strain of TpeRha single-point mutant E462A	This study
*All other single-point mutant strains were constructed in this study and their descriptions were similar to mutant strain BL21 (DE3)/pET-28a-E462A, except for the different mutation site		
BL21 (DE3)/pET-28a-DH	BL21(DE3) engineered strain of TpeRha double-point mutant DH, which mutated both D506 and H570 to alanine	This study
*All other double-point mutant strains were constructed in this study and their descriptions were similar to mutant strain BL21 (DE3)/pET-28a-DH, except for the different mutation site		
BL21 (DE3)/pET-28a-DHK	BL21(DE3) engineered strain of TpeRha triple-point mutant DHK, which simultaneously mutated D506, H570 and K579 to alanine	This study
Plasmids		
pET-28a-TpeRha	Expression vector, pET-28a with the gene *TpeRha* under P_T7_ promoter	General Biosystems Co., Ltd. (Anhui, China)

### Recombinant Protein Expression and Purification

The plasmid pET-28a-TpeRha was transformed into *E. coli* BL21 (DE3). The recombinant TpeRha expression strains were cultured overnight in 5 ml of Luria-Bertani (LB) medium [1% (w/v) tryptone, 0.5% (w/v) yeast extract, 1% (w/v) NaCl, pH 7.4] contained kanamycin (100 μg/ml) and then was inoculated with 1% seed solution into 100 ml fresh Terrific Broth (TB) medium [1.2% (w/v) tryptone, 2.4% (w/v) yeast extract, 0.4% (v/v) glycerol, 17 mM KH_2_PO_4_, 72 mM K_2_HPO_4_] contained kanamycin (100 μg/ml) at 37°C with mixing at 200 rpm for 2, 3 h until the optical density at 600 nm (OD_600_) reached 0.6–0.8. Then, IPTG was added at a final concentration of 0.5 mM to induce gene expression, and the cell cultures were incubated for an additional 16 h at the same condition. The following operations were performed on ice or at 4°C unless otherwise stated. After induction, cells were harvested by centrifugation (5,000 rpm, 4°C, 10 min) and washed twice with phosphate-buffered saline (PBS) [137 mM NaCl, 2.7 mM KCl, 10 mM Na_2_HPO_4_, and 2 mM KH_2_PO_4_, pH7.4]. The precipitates were resuspended in 10 ml lysis buffer [PBS buffer (pH 7.4), 1 mM PMSF], then sonicated for 12 min in an ice bath with 25% intermittent power (4 s on, 6 s off) by Ultrasonic homogenizer SCIENTZ-ⅡD (Ningbo Scientz Biotechnology Co., Ltd., China). The sample was centrifuged at 8,000 rpm at 4°C for 15 min to separate crude extracts and cellular debris. The crude extract was filtered through a 0.45 μm membrane and partially purified by affinity chromatography in Ni-NTA Beads 6FF agarose (Changzhou Smart-Life Sciences Biotechnology Co., Ltd., China) according to the manufacturer’s instructions. The final purified protein removed imidazole and was concentrated *via* ultrafiltration (50 kDa, Millipore, Billerica, MA, United States). The purified protein was detected by sodium dodecyl sulfate-polyacrylamide gel electrophoresis (SDS-PAGE) (12%) followed by Coomassie blue staining. The concentration of protein was determined with Bradford Protein Assay Kit (Takara Biomedical Technology Co., Ltd., China).

### Characterization and Kinetics of the Recombinant TpeRhas

The recombinant TpeRha optimal temperature was determined in a total reaction volume of 500 μl containing 1 mg/ml icariin and 0.05 mg/ml TpeRha in 100 mM disodium hydrogen phosphate-citrate buffer (0.2 M Na_2_HPO_4_ and 0.1 M citric acid, pH 4.6). The reaction was incubated under the temperature range of 55°C–90°C for 4 h.

The recombinant TpeRha optimal pH was determined in a total reaction volume of 500 μl containing 1 mg/ml icariin and 0.05 mg/ml TpeRha in 100 mM disodium hydrogen phosphate-citrate buffer with pH ranging from 4.0 to 7.0. The reactions were incubated under the temperature of 80°C for 4 h.

Reactions were terminated by adding an equal volume of dimethyl sulfoxide (DMSO) and analyzed by high-performance liquid chromatography (HPLC) [Xtimate^®^ C18 (4.6 mm × 250 mm, 5 μm), flow rate: 1 ml/min, column temperature: 30°C, DAD detection wavelength: 270 nm, mobile phase: acetonitrile (A)−0.5% acetic acid (B). Gradient elution conditions: from A: B = 30:70 to A: B = 50:50 over 10 min, and then eluted to A: B = 85:15 for another 10 min, and finally returned to the initial condition A: B = 30:70 over 5 min, more details were shown in Supplementary Table S2] after filtered through a 0.45 μm membrane. The conversion rate was calculated as the product peak area/(substrate peak area + product peak area) ×100%.

The kinetic parameters of TpeRha were determined against icariin in a concentration range of 0.1–4 mg/ml under the optimal conditions *via* a proportional weighted fit using a nonlinear regression analysis program based on Michaelis-Menten enzyme kinetics. The product yield was calculated according to the F factor of the standard. All assays were carried out in triplicate.

The purifications, analytical methods, optimal conditions determination, and kinetic data determinations of mutants constructed next were performed by the same operation.

### *In Silico* Studies and Computer-Aided Design

The three-dimension (3D) structure model of TpeRha was predicted by homology modeling *via* MODELLER software (version 10.1) (https://salilab.org/modeller/) in this study (Webb and Sali, 2016). Ranked the sequence identity of the candidate modeling templates by the build_profile module of MODELLER. The structure of α-L-rhamnosidase from *D. thermophilum* was chosen to build the homology model of TpeRha by multiple-modules of MODELLER. The stereochemical property of the 3D model was then evaluated by PROCHECK (Laskowski et al., 1993) with a Ramachandran plot in UCLA-DOE LAB - SAVES v6.0 (https://saves.mbi.ucla.edu/). TpeRha structure was then modified by adding polar hydrogens and charges by AutoDock Tools (https://autodock.scripps.edu) for molecular docking preparation.

The structure of icariin was provided from ZINC (Sterling and Irwin, 2015), a free database of commercially-available compounds for virtual screening (http://zinc15.docking.org/). Icariin structure was then energy minimized and set all its torsional bonds free by AutoDock Tools as well. Molecular docking simulation of TpeRha and icariin was performed *via* AutoDock Vina software (Trott and Olson, 2010; Eberhardt et al., 2021). The docking results were ranked according to the binding affinity between ligand and receptor. All data of results were obtained by analysis using PyMOL software (www.pymol.org). Several candidate residues were chosen for further mutation experiments and virtual mutation studies (Guex and Peitsch, 1997) (Swiss PDB Viewer, https://spdbv.unil.ch/). The docking analysis of mutants were performed by the same operation.

### Site-Directed Mutagenesis

The primers for site mutation are listed in Supplementary Table S2. The plasmid pET-28a-TpeRha was employed as a template, whole-plasmid amplification polymerase chain reaction (PCR) was performed using PrimeSTAR^®^ Max DNA polymerase. The PCR amplification protocol consisted of denaturation at 98°C for 3 min followed by 30 cycles of denaturation at 98°C for 10 s, annealing at 62°C for 15 s, extension at 72°C for 2 min 30 s, and a final hold for an additional 10 min at 72°C. The PCR product was purified and digested at 37°C for 1 h with *Dpn*Ⅰ to remove the template plasmid and then was transformed into *E. coli* DH5α. Plasmids extracted from positive transformants were confirmed by sequencing and transformed into *E. coli* BL21 (DE3). Positive recombinant mutants were cultured as described for induction expression.

### Whole-Cell Primary Screening System

The incubation system was adjusted from 100 to 20 ml, other operations of strain recovery and induction were the same as described above. After induction, cells were harvested and resuspended in PBS buffer. A reaction mixture of 1 ml disodium hydrogen phosphate-citrate buffer (pH 4.6) contained 1 mg/ml icariin and cells with a 40 mg/ml final cell concentration. The mixture was statically incubated at 55°C for 4 h to investigate the whole-cell catalysis function of cells. The reaction mixture was terminated by adding two volumes of DMSO, and then centrifuged at 12,000 rpm for 10 min. The supernatant was analyzed by HPLC. For experimental control, whole-cell catalysis by the cells carrying the empty pET-28a without gene *TpeRha* was performed at the same condition described above. The conversion rate was calculated as described above. All assays were carried out in triplicate.

### Substrate Specificity Analysis

To investigate the substrate specificity of the wild-type enzyme and one of the single-point mutants, several rhamnosyl natural products (e.g., epimedin C, naringin, rutin, hesperidin, NDHC, and icariin) were used as substrates, and whole-cell catalysis experiments were performed as described above. After incubation at the optimal temperature for 4 h, two volumes of DMSO were added to terminate the reaction. All products were detected by HPLC [Xtimate^®^ C18 (4.6 mm × 250 mm, 5 μm), flow rate: 1 ml/min, column temperature: 30°C, DAD detection wavelength: 250 nm (270 nm for icariin and icariside I), mobile phase: the conditions of each product were shown in Supplementary Tables S2–S6]. The specificities of the enzymes were evaluated by analyzing the conversion rate of each product by HPLC results. All assays were carried out in triplicate.

## Results

### Three-Dimensional Predicted Model of TpeRha

**FIGURE 1:**
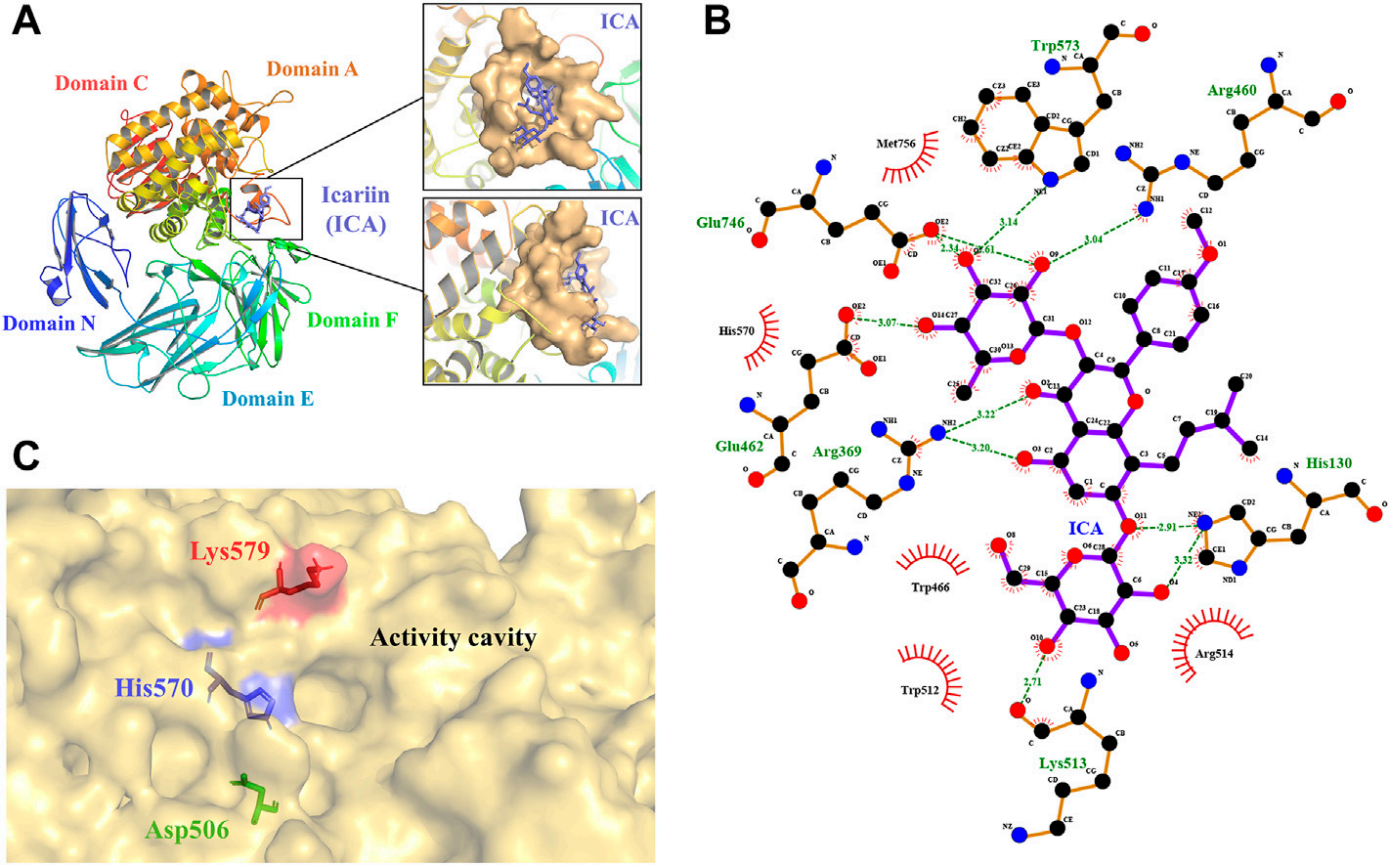
3D model of TpeRha and docking result of TpeRha and icariin. **(A)** The overall fold of the TpeRha model. Five distinct domains are shown in different colors (Domain N-blue, Domain E-cyan, Domain F-green, Domain A-orange, and Domain C-red). The LBP of the TpeRha is shown in the magnified area, the LBP displayed as the surface type shown in bright orange, and the ligand icariin (ICA) displayed as the sticks type shown in purple. **(B)** The receptor-ligand interactions formed between TpeRha and icariin. The hydrogen bonds (H-bonds) are shown as green dashed lines, and the bond lengths are also shown in the schematic. The names of the residues hydrogen-bonded to icariin are shown in green, and the names of nearby residues are shown in black. **(C)** Three residues were selected that could adjust the size of the active cavity based on the distribution of residues near the active pocket. The D506 at the bottom of the pocket is shown in green, the H570 at the edge of the pocket is shown in blue, and the K579 at the surface of the pocket is shown in red.

To explore the relationship between the structure and function of TpeRha and then improve the catalytic efficiency for its designated substrate, a computer-aided protein design strategy was used in this study. Three-dimensional structure prediction was carried out *via* homology modeling, and substrate-enzyme binding state prediction was carried out *via* molecular docking technology. Based on the sequence alignment results of template search in homology modeling, the DtRha (Guillotin et al., 2019) from *D. thermophilum* with the highest sequence identity (48.28%) to TpeRha was selected as the template for modeling (Supplementary Figure S1). The predicted model was assessed by the Ramachandran plot, indicating that the model was reasonable, with 87.8% of the structure in the most favored regions, and only 0.4% of the residues in the disallowed regions (Supplementary Figure S2). The result verified in PROCHECK confirmed the high quality of the model. According to the DtRha structure analysis, the TpeRha structure was predicted to consist of five distinct domains, namely, domain N (1–95), domain E (96–278), domain F (307–397), catalytic domain A (398–754), and domain C (755–861) (Figure 1A).

### Selection of Mutation Sites Based on Molecular Docking Analysis

The results of the docking simulation showed that the designated substrate icariin was bound into the catalytic pocket on the surface of TpeRha. The rhamnosyl group of icariin was precisely located in the active hole. The residues within a 5 Å distance from icariin were defined as an active pocket (Figure 1A), which included His130, Arg369, Asp456, Arg460, Asp461, Glu462, Trp466, Asp506, Val507, Trp512, Lys513, Arg514, Trp521, Gln 569, His 570, Trp573, Cys574, Lys579, Phe580, Glu746, Gly755, Met756, His761, and Met763. According to the analysis, the residues Glu462 and Glu746 of TpeRha might be the catalytic acid and the catalytic base respectively. Moreover, icariin also exhibited interactions with these two residues through H-bonds (Figure 1B). Besides, residues His130, Arg369, Arg460, Lys513, and Trp573 in the active hole were also involved in other H-bond formation (Figure 1B). All residues identified above were mutated to alanine to investigate their effects on enzyme activity.

The binding affinity of the docking complex TpeRha-icariin was −5.7 kcal/mol. This value implies that the binding strength of icariin to TpeRha is weak. Furthermore, the catalytic efficiency of TpeRha for the hydrolysis of icariin to icarside I might be low. Based on the analysis of the docking complex conformation, it was preliminarily speculated that the reason for the high binding affinity may be a steric hindrance from the unique core structure of icariin. In order to expand the cavity capacity of the active pocket, Asp506, His570, and Lys579 were figured out for mutating to alanine (Figure 1C), according to the rules including suitable residues located at the bottom of the active pocket and at the edge of the active pocket.

### Whole-Cell Catalysis Screening Results

**FIGURE 2:**
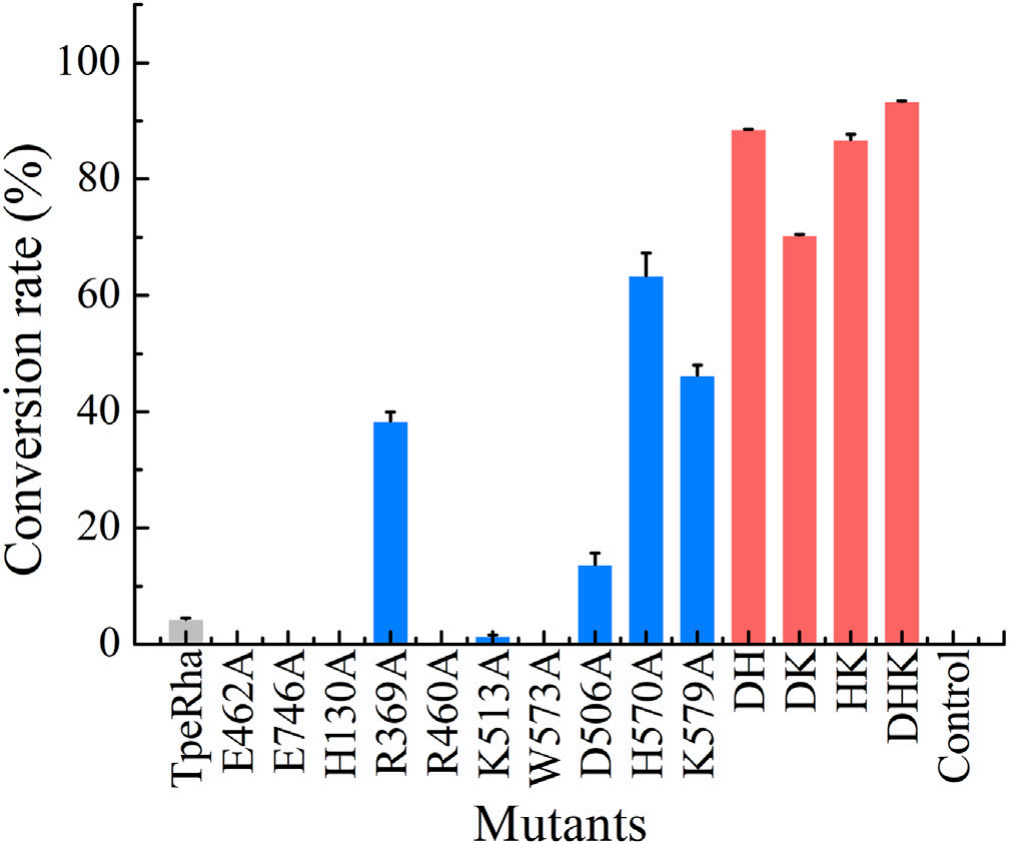
The whole-cell catalysis screening results of mutants designed in this study are shown in three colored bars (wild-type shown in light gray, single-point mutants shown in blue, and multiple-point mutants shown in red). The conversation rate of the wild-type TpeRha was 4.33% ± 0.19%, while the control was product undetected. DHK had the highest conversion rate of 93.32% ± 0.09%.

Construction of mutants including E462A, E746A, H130A, R369A, R460A, K513A, W573A, D506A, H570A, and K579A was chieved *via* whole-plasmid amplification PCR. All mutants together with the wild-type TpeRha and the control strain were incubated and induced for enzyme expression. Cells were collected and added to the whole-cell catalytic reaction system. The results showed that the conversion rate of icariin to icariside I by the wild-type TpeRha was 4.33% ± 0.19%, and no icariside I was detected in the control group (Figure 2). As expected, E462A, E746A, H130A, R460A, and W573A lost their hydrolytic activity for icariin. The activity of K513A was greatly reduced, only 1.41% ± 0.21%. Surprisingly, the activity of R369A mutant increased to 38.37% ± 1.57%, 8.86 times higher than that of the wild-type TpeRha. As for the three rational designed mutants D506A, H570A, and K579A, the activities of which were all improved compared with the wild-type TpeRha, the conversion rates reached 13.70% ± 1.93%, 63.65% ± 3.96%, and 46.29% ± 1.78%, respectively (Figure 2). Furthermore, combinatorial mutations of R369A, D506A, H570A, and K579A were carried out. Interestingly, the enzymatic activity was lost when Arg369 was combined with any of the other residues, while the mutual combination of the remaining three mutants resulted in better conversion rates than the corresponding single mutants. The conversion rates of the combinatorial mutants DH (D506A/H570A), DK (D506A/K579A), HK (H570A/K579A), and DHK (D506A/H570A/K579A) were 88.56% ± 0.06%, 70.38% ± 0.13%, 86.78% ± 0.90%, and 93.32% ± 0.09%, respectively (Figure 2).

### Enzymatic Characterization of TpeRhas

**FIGURE 3:**
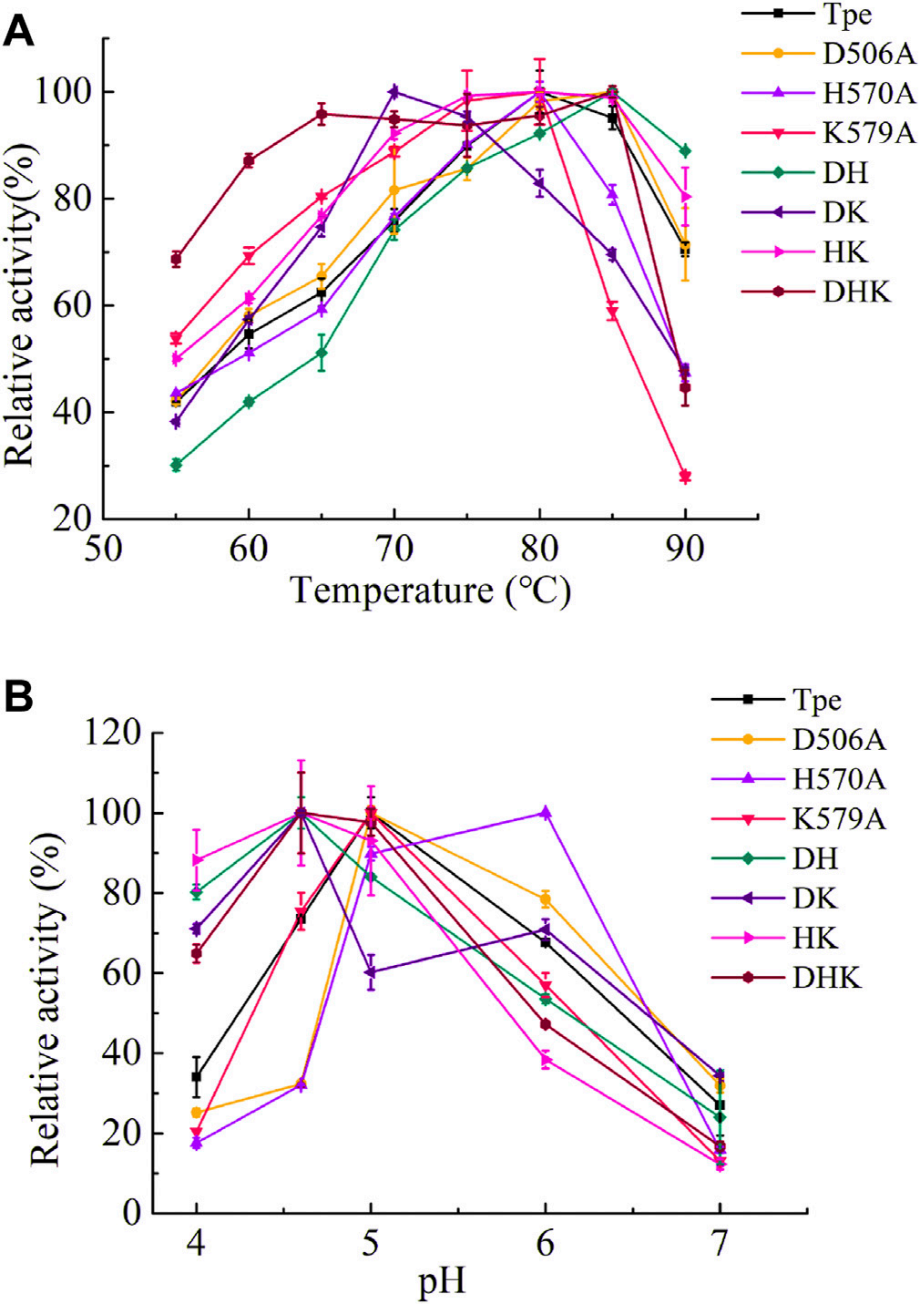
The optimal conditions of TpeRha and mutants. **(A)** The optimal temperatures of TpeRha and mutants. **(B)** The optimal pH values of TpeRha and mutants.

The wild-type TpeRha and the beneficial mutants were purified by affinity chromatography with Ni-NTA Beads 6FF agarose, and the purified enzymes were assessed for their catalytic activities. The optimal temperature of the TpeRha was 80°C, and that of the mutants H570A, K579A, and HK were the same as the TpeRha, while the optimal temperature of D506A, DH, and DHK were 85°C (Figure 3A). However, the mutant DK was more suitable for catalysis at a relatively lower temperature of 70°C. Among them, the mutant DHK kept a high enzymatic activity (above 90%) in the range of 65°C to 85°C. The optimal pH of TpeRha was 5.0, and the mutants D506A and K579A had the same as TpeRha. The optimal pH of mutant H570A was 6.0, while that for DH, DK, HK, and DHK was 4.6 (Figure 3B). All enzymes here required an acidic condition for catalysis.

Fitting the kinetic data obtained for the wild-type TpeRha with the substrate icariin into non-linear regression yielded a *V*_*max*_ of 0.098 ± 0.054 μM/min and a *K*_*M*_ of 4.33 ± 3.47 μM for icariin. The detailed kinetic parameters of the TpeRha and mutants were determined using icariin as the substrate and are listed in Table 2 (Supplementary Figure S3). As shown in Table 2, the catalytic efficiency (*k*_*cat*_/*K*_*M*_) of all mutants against icariin significantly surpassed that of the wild-type TpeRha. The *k*_*cat*_/*K*_*M*_ values of single-point mutants D506A, H570A, and K579A were 16.12 times, 42.84 times, and 37.65 times higher than that of the wild-type TpeRha, respectively. Furthermore, double-point mutants exhibited more significant catalytic efficiency than that of single-point mutants. The *k*_*cat*_/*K*_*M*_ values of double-point mutants DH, DK, and HK were 209.76 times, 44.30 times, and 137.69 times higher than that of the wild-type TpeRha. However, the triple-point mutant DHK did not show a superior effect to the double-point mutation but was slightly lower than that of the double-point mutant DH. The catalytic efficiency of DHK was 206.60 times higher compared with the wild-type TpeRha.

**TABLE 2:** Kinetic parameters of the TpeRha mutants against icariin.

TpeRhas	*Vmax* (μM·min^−1^)	*K*_*M*_ (μM)	*kcat* (s^−1^) (×10^−4^)	*kcat*/*K*_*M*_ (s^−1^·M^−1^)
Wild-type	0.098 ± 0.054	4.33 ± 3.47	0.040	0.922569
D506A	1.906 ± 0.124	3.12 ± 0.26	0.464	14.86935
H570A	1.191 ± 0.044	0.92 ± 0.08	0.364	39.52007
K579A	1.968 ± 0.120	1.24 ± 0.09	0.431	34.73855
DH	2.505 ± 0.299	0.51 ± 0.13	0.987	193.5216
DK	1.369 ± 0.250	1.39 ± 0.45	0.568	40.86892
HK	5.564 ± 0.203	0.93 ± 0.08	1.181	127.0272
DHK	6.592 ± 0.856	1.11 ± 0.34	2.116	190.6003

### Docking Analysis of Mutants

**FIGURE 4:**
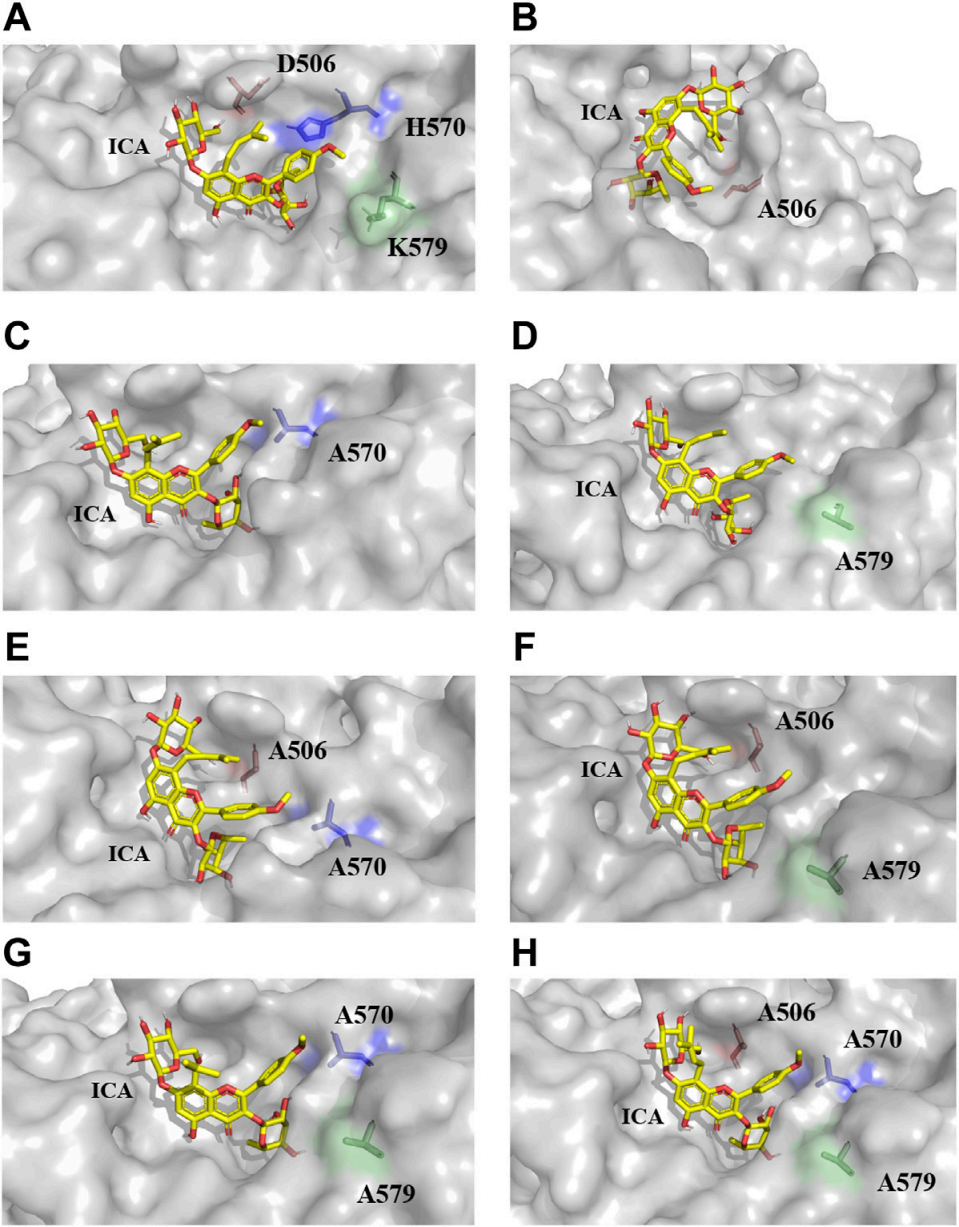
The docking results of TpeRhas and icariin. The TpeRhas models are displayed as the surface type shown in gray, the icariin is displayed as stick type shown in yellow, the residue at position 506 is displayed as sticks type shown in salmon, the residue at position 570 is displayed as stick type shown in blue, and the residue at position 579 is displayed as stick type shown in green. **(A)** The conformation of docking complex TpeRha-icariin. **(B)** The conformation of docking complex D506A-icariin. **(C)** The conformation of docking complex H570A-icariin. **(D)** The conformation of docking complex K579A-icariin. **(E)** The conformation of docking complex DH-icariin. **(F)** The conformation of docking complex DK-icariin. **(G)** The conformation of docking complex HK-icariin. **(H)** The conformation of docking complex DHK-icariin.

All models of mutants were constructed *via* Swiss PDB Viewer (Guex and Peitsch, 1997). The results output by Autodock Vina included binding affinity and number of hydrogen bonds, which reflect the combinational effects of steric hindrance, flexibility, interaction force and other relevant factors. The binding affinity of the mutants and icariin complexes was lower than that of the wild-type TpeRha (Table 3). The docking results and schematic results of receptor-ligand interaction are shown in Figure 4 and Supplementary Figure S4, respectively. As the binding affinity of these docked complexes and the hydrogen bonding force formed between the mutants and the icariin somehow reflected the difficulty of catalytic reaction, lower binding affinity indicated conformations of the mutant complexes were more stable. Therefore, the reaction was more efficient by using a mutant TpeRha. However, the interaction force between the wild-type TpeRha and icariin was the strongest, in which 10 hydrogen bonds were formed (Figure 1B). Decreased hydrogen bonds between the mutants and icariin might have resulted from the shortening of the side chain groups of the mutational amino acids. Although the docking complex H570A-icariin showed the lowest binding affinity (−7.7 kcal/mol) among all complexes, the interaction force between H570A and icariin was the weakest, with only five hydrogen bonds were formed (Supplementary Figure S3C). The complex with the least influence on hydrogen bonding force and the relatively most stable conformation was the DH-icariin complex (nine hydrogen bonds and −6.5 kcal/mol binding affinity) (Supplementary Figure S3E), which implied that DH was the receptor with the best binding mode for icariin in all mutants. Furthermore, this result of docking analysis was consistent with the result of the kinetic characterization that DH was the best mutant with the highest catalytic efficiency; 209.76 times higher compared with the wild-type TpeRha.

**TABLE 3:** Parameters affecting the docking of mutants and icariin complexes.

TpeRhas	Binding affinity (kcal/mol)	Numbers of hydrogen bonds
Wild-type	−5.7	10
D506A	−6.2	8
H570A	−7.7	5
K579A	−6.3	8
DH	−6.5	9
DK	−6.4	9
HK	−6.3	8
DHK	−7.6	5

### Substrate Specificity Analysis

**FIGURE 5:**
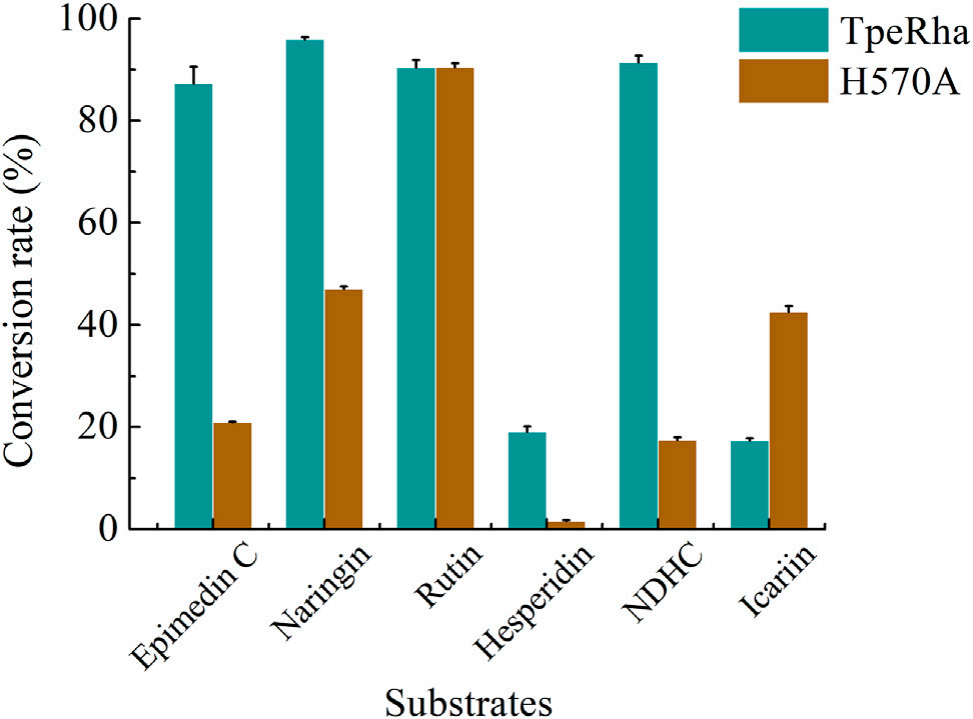
The conversion rates of TpeRha and H570A reacted with different substrates. The conversion rates of epimedin C, naringin, hesperidin, and NDHC for TpeRha were higher than that for H570A. The conversion rates of the substrate rutin for both were comparable. The conversion rate of icariin for H570A was 2.45 times higher than that for TpeRha.

Icariin, epimedin C, naringin, rutin, hesperidin, and NDHC were chosen for substrate specificity on the wild-type TpeRha and the single-point mutant H570A (Supplementary Figure S5–S10). When icariin was used as the substrate, the conversion rate of producing icariside I by the mutant H570A was higher than that of the wild-type TpeRha (Figure 5). Furthermore, compared with the wild-type TpeRha, the catalytic efficiencies of mutant H570A were lower when using epimedin C, naringin, hesperidin, and NDHC as substrates. The activity was basically unchanged when rutin was used as the substrate. These results indicated that the substrate specificity of mutant H570A for icariin was improved.

## Discussion

α-L-rhamnosidase is a specific enzyme for hydrolyzing natural products with the rhamnosyl group (Manzanares et al., 2007). This study aimed to develop an efficient α-L-rhamnosidase for hydrolyzing the rhamnosyl group of icariin to produce icariside I. TpeRha from *T. petrophila* DSM 13995 (Xie et al., 2020) is one of the enzymes that has the hydrolytic activity for icariin to icariside I. Both Rhase-I from *T. stollii* CLY-6 (Cheng et al., 2022) and DthRha from *D. thermophilum* DSM 3960 (Zhang et al., 2021) have also been reported for the hydrolysis of the rhamnosyl group. However, the 3D structure of Rhase-I is not currently available, and homology modeling cannot be performed since the enzyme with the highest protein sequence identity with Rhase-I is only at 25.20% (PDB ID: 6q2f) (Mensitieri et al., 2018). Cheng et al. (2022) used an improved deep learning-based modeling method, RoseTTAFold, to predict the Rhase-I 3D structure, and performed docking studies using the DOCK 6.9 program. According to the results of the study mentioned above, this enzyme was excluded in this study after careful evaluation, due to lack of a report on hydrolyzation of icariin to generate icariside I by its model template protein Rha-P (PDB ID: 6q2f). Rational design with undefined factors would be difficult. Fortunately, the 3D structure of DthRha can be availed from the PDB database. Interestingly, in a study of structure determination by X-ray diffraction (Guillotin et al., 2019), DthRha was not reported for its activity of hydrolyzing icariin to generate icariside I. This enzyme was later used in icaritin synthesis by Zhang et al. (2021). Therefore, TpeRha, which was first reported to have the activity of hydrolyzing icariin to generate icariside I, was selected as the target protein in this study.

**FIGURE 6:**
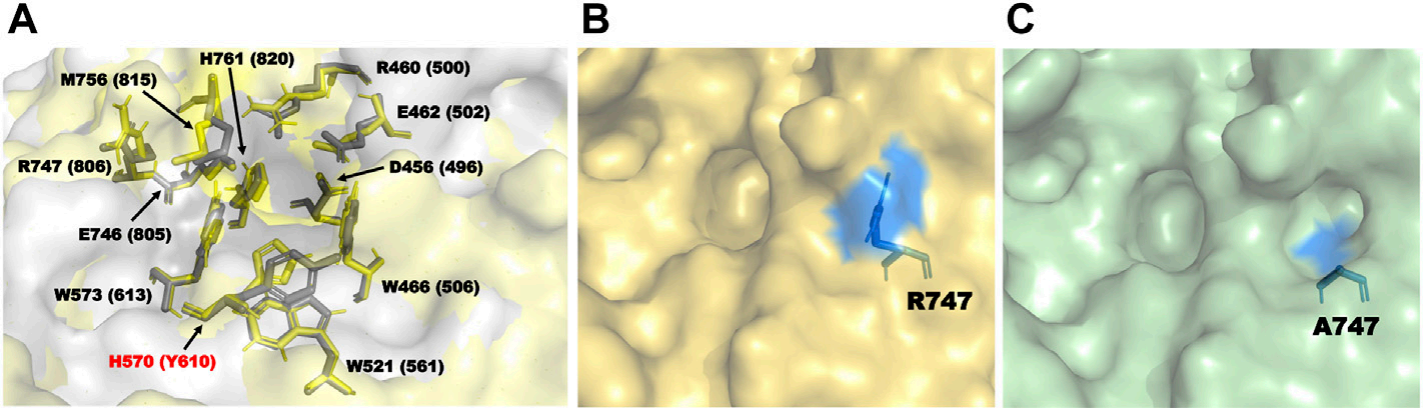
The key residues in the TpeRha model and DtRha model. **(A)** Superimposition of active sites of TpeRha built in this study and DtRha from *D. thermophilum*. Residues are respectively colored in yellow and gray. Numbers indicate the residue from TpeRha and the corresponding residues from DtRha. The conflict residue is H570 of TpeRha and Y610 of DtRha. **(B)** The location and status of residue R747 in TpeRha. **(C)** The location and status of residue A747 in TpeRha.

During homology modeling of TpeRha, the template with the highest sequence identity (48.28%) was DthRha (PDB ID: 6i60) (Guillotin et al., 2019). By aligning the 3D structure of TpeRha obtained by homology modeling with the 3D structure of template DthRha, we figure out the active pocket of TpeRha. According previous study on DthRha, there are 11 amino acids encapsulated in rhamnose; D496, R500, E502, W506, W561, Y610, W613, E805, R806, M815, and H820. Interestingly, these corresponding amino acids were basically found in the active pocket of TpeRha, and the matching degree was as high as 90.9%. Only the amino acid H570 was different, and was considered as a key design target in this study (Figure 6A).

In addition to improvement of catalytic efficiency, this study also designed a mutant, H570Y, to explore whether it could maintain or change the catalytic activity of icariin after mimicking the DthRha active pocket in TpeRha. The conversion rate of icariin by mutant H570Y was 4.25 times higher than that of the wild-type, reaching 18.41% ± 2.05%, but it was still lower than that of the mutant H570A (63.65% ± 3.96%). Based on these results, we proposed the hypothesis that DthRha also has the activity of hydrolyzing icariin, which was in agreement with results reported by Zhang et al. (2021).

Nine amino acids of the 10 identified key amino acids were confirmed for their importance to the function of TpeRha. No icariside I was produced in whole-cell catalysis with 9 TpeRha mutants (D456A, R460A, E462A, W466A, W521A, W573A, E746A, M756A, and H761A). The conversion rate of the mutant R747A was increased by 3.07 times (13.31% ± 0.21%). Based on the molecular docking result, R747 located at the border of the active pocket, and the distance between icariin and R747 was 6.8 Å, which was beyond generating an interaction force. Thus, R747 might not be involved in the function of locking the rhamnosyl group. After mutating arginine to alanine at the 747-site, the side chain group became shorter, and a depression was formed on the surface of the protein (Figures 6B,C). It was speculated that this change increased the probability of icariin binding to the active pocket, and resulted in the conversion rate improvement.

Based on the conformation structure of the docking complex TpeRha-icariin, the poor conformational stability of this complex may be due to the p-methoxybenzene ring on the core structure of icariin (Figure 4A). The non-rotatability of the p-methoxybenzene ring might result in a rigid steric hindrance, which hindered the rhamnose group entering the active pocket. In order to offer the rhamnosyl group of icariin easy access to the active pocket of the enzyme, potential steric hindrance was reduced on purpose. Three non-critical amino acids were rationally designed, which were located inside the enzyme (the bottom of the active pocket), at the active pocket border, and the active pocket surface, respectively. D506, located inside the active pocket, was mutated to alanine to increase the depth of the active pocket. H570, located at the active pocket border, was mutated to alanine to increase the caliber of the active pocket. K579, located at the surface of the active pocket, was mutated to alanine to flatten the active pocket surface (Figure 1C). As expected, the catalytic efficiencies of these three mutants were improved, showing a useful strategy for improvement by increasing the caliber of the active pocket. The catalytic efficiency of H570A was 42.84 times higher than that of the wild type. This suggested that reducing the steric hindrance between the active pocket and icariin was an effective strategy to improve the catalytic efficiency of this enzyme towards icariin. The results of the protein-ligand interaction showed that the number of hydrogen bonds between the mutants and icariin was less than that between the wild-type and icariin, which suggested that the larger the active pocket of the cavity, the weaker the interaction force between the substrate and the enzyme. This may explain why the catalytic efficiency of the triple-point mutant DHK was less efficient than that of the double-point mutant DH (Table 2).

Furthermore, to the best of our knowledge, most α-L-rhamnosidases are sensitive to α-1, 2, and α-1, 6 glycosidic bonds (Manzanares et al., 2007). Only a small part of α-L-rhamnosidase has the function of cleaving the glycosidic bond α-1, while the enzymes hydrolyzing the glycosidic bond α-1 of icariin are only the three we mentioned above in this study. The reason may be that the small volume of the active cavity in the core structure hinders the rhamnosyl group connected by the glycosidic bond α-1 to the active pocket. Intentionally, in this study, the mutant H570A that only expanded the caliber of the active pocket was used to hydrolyze the rhamnosyl group of a variety of substrates, whose rhamnosyl linkages covered α-1, α-1, 2, and α-1, 6, to investigate whether changes in the size of the active pocket could affect substrate specificity. Except for icariin with glycosidic bond α-1, the conversion rates of all other substrates with glycosidic bond α-1, 2, and α-1, 6 were reduced. This implied that the enlarged caliber of the active pocket partly loosened the ‘lock’ of the rhamnosyl group. In turn, this designed mutation improved the substrate specificity for icariin.

## Conclusion

In conclusion, the active pocket and key residues of the TpeRha from *T. petrophila* DSM 13995 were revealed *via* computer-aided technologies, like homology modeling and molecular docking, in this study. Several efficient mutants of α-L-rhamnosidases were rationally designed for hydrolyzing the icariin to produce icariside I The double-point mutant DH was the most beneficial mutant and showed the highest catalytic efficiency (*k*_*cat*_/*K*_*M*_: 193.52 s^−1^ M^−1^) against icariin, which was a 209.76-fold increase compared with the wild-type TpeRha. Meanwhile, a single-point mutant H570A with high substrate specificity was also obtained in this study. In addition, the strategy of adjusting the size of the active cavity led to many interesting discoveries which explain why there are currently only three wild-type α-L-rhamnosidases that can hydrolyze icariin. This study also provides strategies and principles for designing enzymes for hydrolyzing compounds with special core structures.

## Data Availability

The original contributions presented in the study are included in the article/Supplementary Material, further inquiries can be directed to the corresponding authors.

## Abbreviations

3D, three-dimensional; DH, TpeRha double-point mutant, which mutated both D506 and H570 to alanine; DK, TpeRha double-point mutant, which mutated both D506 and K579 to alanine; DHK, TpeRha triple-point mutant, which simultaneously mutated D506, H570 and K579 to alanine; HK, TpeRha double-point mutant, which mutated both H570 and K579to alanine; HPLC, high-performance liquid chromatography; IPTG, Isopropyl-β-D-thiogalactopyranoside; LB, Luria–Bertani; TB, Terrific Broth; SDS-PAGE, sodium dodecyl sulfate-polyacrylamide gel electrophoresis.

## References

[B1] Chen G. Cao Z. Shi Z. Lei H. Chen C. Yuan P. (2021). Microbiome Analysis Combined with Targeted Metabolomics Reveal Immunological Anti-tumor Activity of Icariside I in a Melanoma Mouse Model. Biomed. Pharmacother. 140, 111542. 10.1016/j.biopha.2021.111542 34088571

[B2] Cheng L. Zhang H. Cui H. Cheng J. Wang W. Wei B. (2022). A Novel α-L-Rhamnosidase Renders Efficient and Clean Production of Icaritin. J. Clean. Prod. 341, 130903. 10.1016/j.jclepro.2022.130903

[B3] Eberhardt J. Santos-Martins D. Tillack A. F. Forli S. (2021). AutoDock Vina 1.2.0: New Docking Methods, Expanded Force Field, and python Bindings. J. Chem. Inf. Model. 61 (8), 3891–3898. 10.1021/acs.jcim.1c00203 34278794 PMC10683950

[B4] Guex N. Peitsch M. C. (1997). SWISS-MODEL and the Swiss-Pdb Viewer: An Environment for Comparative Protein Modeling. Electrophoresis 18 (15), 2714–2723. 10.1002/elps.1150181505 9504803

[B5] Guillotin L. Kim H. Traore Y. Moreau P. Lafite P. Coquoin V. (2019). Biochemical Characterization of the α-l-Rhamnosidase DtRha from Dictyoglomus Thermophilum: Application to the Selective Derhamnosylation of Natural Flavonoids. ACS Omega 4 (1), 1916–1922. 10.1021/acsomega.8b03186 31459445 PMC6649072

[B6] Huang X. Zhu D. Lou Y. (2007). A Novel Anticancer Agent, Icaritin, Induced Cell Growth Inhibition, G1 Arrest and Mitochondrial Transmembrane Potential Drop in Human Prostate Carcinoma PC-3 Cells. Eur. J. Pharmacol. 564 (1-3), 26–36. 10.1016/j.ejphar.2007.02.039 17382317

[B7] Huang J. J. Wei T. Ye Z. W. Zheng Q. W. Jiang B. H. Han W. F. (2021). Microbial Cell Factory of Baccatin Ⅲ Preparation in *Escherichia coli* by Increasing DBAT Thermostability and *In Vivo* Acetyl-CoA Supply. Front. Microbiol. 12, 803490. 10.3389/fmicb.2021.803490 35095813 PMC8790024

[B8] Khan F. I. Wei D.-Q. Gu K.-R. Hassan M. I. Tabrez S. (2016). Current Updates on Computer Aided Protein Modeling and Designing. Int. J. Biol. Macromol. 85, 48–62. 10.1016/j.ijbiomac.2015.12.072 26730484

[B9] Laskowski R. A. MacArthur M. W. Moss D. S. Thornton J. M. (1993). PROCHECK: A Program to Check the Stereochemical Quality of Protein Structures. J. Appl. Cryst. 26, 283–291. 10.1107/s0021889892009944

[B10] Li W.-K. Zhang R.-Y. Pei-Gen X. (1996). Flavonoids from Epimedium Wanshanense. Phytochemistry 43 (2), 527–530. 10.1016/0031-9422(96)00187-2 8862041

[B11] Li J. Liu P. Zhang R. Cao L. Qian H. Liao J. (2011). Icaritin Induces Cell Death in Activated Hepatic Stellate Cells through Mitochondrial Activated Apoptosis and Ameliorates the Development of Liver Fibrosis in Rats. J. Ethnopharmacol. 137 (1), 714–723. 10.1016/j.jep.2011.06.030 21726622

[B12] Li L. J. Wu Z. Y. Yu Y. Zhang L. J. Zhu Y. B. Ni H. (2018). Development and Characterization of an α-l-rhamnosidase Mutant with Improved Thermostability and a Higher Efficiency for Debittering Orange Juice. Food Chem. 245, 1070–1078. 10.1016/j.foodchem.2017.11.064 29287324

[B13] Li B.-C. Peng B. Zhang T. Li Y.-Q. Ding G.-B. (2019). A Spectrophotometric Method for High-Throughput Screening of α-l-rhamnosidase Activity on Rutin Coupled with a β-d-glucosidase Assay. 3 Biotech. 9 (6), 227. 10.1007/s13205-019-1753-1 PMC6529497 31139542

[B14] Li D.-D. Jiang Y.-P. Wang Z.-Z. Xiao W. Zhao L.-G. (2020). Molecular Insights into Catalytic Specificity of α-L-rhamnosidase from Bacteroides Thetaiotaomicron by Molecular Docking and Dynamics. Chem. Phys. Lett. 754, 137695. 10.1016/j.cplett.2020.137695

[B15] Liao H. Gong J. Y. Yang Y. Jiang Z. D. Zhu Y. B. Li L. J. (2019). Enhancement of the Thermostability of Aspergillus niger α‐ L ‐rhamnosidase Based on PoPMuSiC Algorithm. J. Food. Biochem. 43 (8), e12945. 10.1111/jfbc.12945 31368575

[B16] Lyu Y. Zeng W. Du G. Chen J. Zhou J. (2019). Efficient Bioconversion of Epimedin C to Icariin by a Glycosidase from *Aspergillus nidulans* . Bioresour. Technol. 289, 121612. 10.1016/j.biortech.2019.121612 31203178

[B17] Ma H. He X. Yang Y. Li M. Hao D. Jia Z. (2011). The Genus *Epimedium*: An Ethnopharmacological and Phytochemical Review. J. Ethnopharmacol. 134 (3), 519–541. 10.1016/j.jep.2011.01.001 21215308

[B18] Manzanares P. Vallés S. Ramòn D. Orejas M. (2007). “α-L-rhamnosidases: Old and New Insights,” in Industrial Emzymes. Valencia, Spain: Springer, 117–140.

[B19] Mensitieri F. De Lise F. Strazzulli A. Moracci M. Notomista E. Cafaro V. (2018). Structural and Functional Insights into RHA-P, a Bacterial GH106 α-L-rhamnosidase from Novosphingobium Sp. PP1Y. Archives Biochem. Biophys. 648, 1–11. 10.1016/j.abb.2018.04.013 29678627

[B20] Mu G. Pu W. Zhou M. Liu Y. Yang H. Wang C. (2013). Synthesis of Icaritin. Chin. J. Org. Chem. 33 (6), 1298. 10.6023/cjoc201303016

[B21] Saito Y. Oikawa M. Nakazawa H. Niide T. Kameda T. Tsuda K. (2018). Machine-learning-guided Mutagenesis for Directed Evolution of Fluorescent Proteins. ACS Synth. Biol. 7 (9), 2014–2022. 10.1021/acssynbio.8b00155 30103599

[B22] Sterling T. Irwin J. J. (2015). ZINC 15 - Ligand Discovery for Everyone. J. Chem. Inf. Model. 55 (11), 2324–2337. 10.1021/acs.jcim.5b00559 26479676 PMC4658288

[B23] Trott O. Olson A. J. (2010). AutoDock Vina: Improving the Speed and Accuracy of Docking with a New Scoring Function, Efficient Optimization, and Multithreading. J. Comput. Chem. 31 (2), NA. 10.1002/jcc.21334 PMC3041641 19499576

[B24] Wang P. Li C. Li X. Huang W. Wang Y. Wang J. (2021). Complete Biosynthesis of the Potential Medicine Icaritin by Engineered *Saccharomyces cerevisiae* and *Escherichia coli* . Sci. Bull. 66 (18), 1906–1916. 10.1016/j.scib.2021.03.002 36654400

[B25] Webb B. Sali A. (2016). Comparative Protein Structure Modeling Using MODELLER. Curr. Protoc. Protein Sci. 86, 2 9 1–2 9 37. 10.1002/cpps.20 27801516

[B26] Wu T. Pei J. Ge L. Wang Z. Ding G. Xiao W. (2018). Characterization of a α-l-rhamnosidase from Bacteroides Thetaiotaomicron with High Catalytic Efficiency of Epimedin C. Bioorg. Chem. 81, 461–467. 10.1016/j.bioorg.2018.08.004 30243237

[B27] Xie J. Zhang S. Tong X. Wu T. Pei J. Zhao L. (2020). Biochemical Characterization of a Novel Hyperthermophilic α-l-rhamnosidase from Thermotoga Petrophila and its Application in Production of Icaritin from Epimedin C with a Thermostable β-glucosidase. Process Biochem. 93, 115–124. 10.1016/j.procbio.2020.03.019

[B28] Xu Y. Li Z. Yuan L. Zhang X. Lu D. Huang H. (2013). Variation of Epimedins A - C and Icariin in Ten Representative Populations of Epimedium brevicornu Maxim., and Implications for Utilization. Chem. Biodivers. 10, 711–721. 10.1002/cbdv.201100424 23576357

[B29] Xu L. Liu X. Yin Z. Liu Q. Lu L. Xiao M. (2016). Site-directed Mutagenesis of α-l-rhamnosidase from Alternaria Sp. L1 to Enhance Synthesis Yield of Reverse Hydrolysis Based on Rational Design. Appl. Microbiol. Biotechnol. 100 (24), 10385–10394. 10.1007/s00253-016-7676-4 27352363

[B30] Yang K. K. Wu Z. Arnold F. H. (2019). Machine-learning-guided Directed Evolution for Protein Engineering. Nat. Methods. 16 (8), 687–694. 10.1038/s41592-019-0496-6 31308553

[B31] Ye M. Han J. Chen H. Zheng J. Guo D. (2007). Analysis of Phenolic Compounds in Rhubarbs Using Liquid Chromatography Coupled with Electrospray Ionization Mass Spectrometry. J. Am. Soc. Mass Spectrom. 18 (1), 82–91. 10.1016/j.jasms.2006.08.009 17029978

[B32] Zhang S. Luo J. Dong Y. Wang Z. Xiao W. Zhao L. (2021). Biotransformation of the Total Flavonoid Extract of Epimedium into Icaritin by Two Thermostable Glycosidases from *Dictyoglomus Thermophilum* DSM3960. Process Biochem. 105, 8–18. 10.1016/j.procbio.2021.03.002

[B33] Zhu D.-y. Lou Y.-j. (2005). Inducible Effects of Icariin, Icaritin, and Desmethylicaritin on Directional Differentiation of Embryonic Stem Cells into Cardiomyocytes *In Vitro* . Acta Pharmacol. Sin. 26 (4), 477–485. 10.1111/j.1745-7254.2005.00076.x 15780198

