# Supplementary material for: Computer-Aided Design of α-L-Rhamnosidase to Increase the Synthesis Efficiency of Icariside I
Source: Front Bioeng Biotechnol. 2022 Jun 21;10:926829. doi: 10.3389/fbioe.2022.926829 (PMC9253678; doi:10.3389/fbioe.2022.926829)
Supplement: Supplementary file 1 [file DataSheet1.docx]

**Supplementary materials**

**Computer-aided design of α-L-rhamnosidase to increase the synthesis efficiency of icariside Ⅰ**

**Jia-jun Huang^1,2^, Hao-xuan Hu^2^, Yu-Jing Lu^2,3^, Ya-Dan Bao^2^, Jin-Lin Zhou^2,*^, Mingtao Huang^1,*^**

^1^School of Food Science and Engineering, South China University of Technology, Guangzhou 510641, China

^2^Golden Health Biotechnology Co., Ltd., Foshan 528225, China

^3^School of Chemical Engineering and Light Industry, School of Biomedical and Pharmaceutical Sciences, Guangdong University of Technology, Guangzhou 510006, China.

***Correspondence:**

Corresponding Author: Prof. Mingtao Huang and Dr. Jinlin Zhou.

E-mails: huangmt@scut.edu.cn (M.T.H.) and david_zhou@goldenhealth.com.cn (J.L.Z.).

| **Fig. S1.** | The sequence alignment of TpeRha and the template DtRha. |
| --- | --- |
| **Fig. S2.** | The Ramachandran plot of TpeRha model. |
| **Fig. S3.** | The kinetic curves of TpeRha and mutants with different concentrations of icariin. (A) TpeRha, (B) D506A, (C) H570A, (D) K579A, (E) DH, (F) DK, (G) HK, (H) DHK. |
| **Fig. S4.** | The schematic results of receptor-ligand interaction between TpeRhas and icariin. (A) TpeRha, (B) D506A, (C) H570A, (D) K579A, (E) DH, (F) DK, (G) HK, (H) DHK. The hydrogen bonds between TpeRhas and icariin were shown as green dotted lines. |
| **Fig. S5.** | The HPLC results of epimedin C hydrolyzed by TpeRha (A) and H570A (B). |
| **Fig. S6.** | The HPLC results of naringin hydrolyzed by TpeRha (A) and H570A (B). |
| **Fig. S7.** | The HPLC results of rutin hydrolyzed by TpeRha (A) and H570A (B). |
| **Fig. S8.** | The HPLC results of hesperidin hydrolyzed by TpeRha (A) and H570A (B). |
| **Fig. S9.** | The HPLC results of NDHC hydrolyzed by TpeRha (A) and H570A (B). |
| **Fig. S10.** | The HPLC results of icariin hydrolyzed by TpeRha (A) and H570A (B). |
| **Table S1** | The pairs of forward and reverse primers used for mutation. |
| **Table S2** | The gradient elution conditions for epimedin C, icariin and icariside Ⅰ analysis |
| **Table S3** | The gradient elution conditions for naringin and naringenin-7-O-glucoside analysis. |
| **Table S4** | The gradient elution conditions for rutin and isoquercetin analysis. |
| **Table S5** | The gradient elution conditions for hesperidin and hesperetin-7-O-glucoside analysis. |
| **Table S6** | The gradient elution conditions for NDHC and trilobatin analysis. |


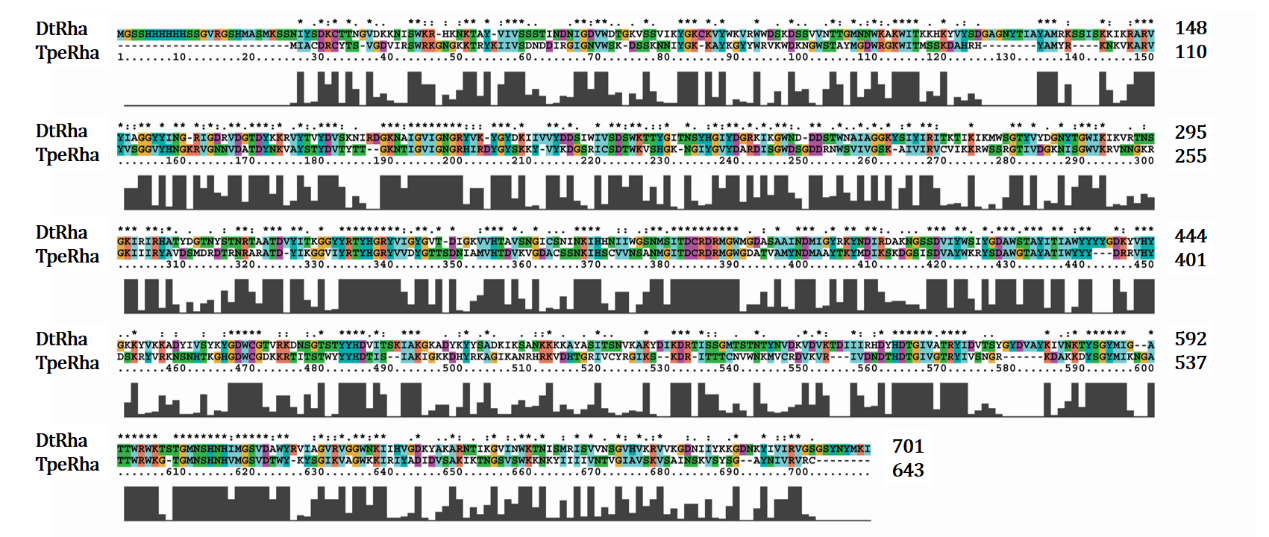


**Fig. S1.** The sequence alignment of TpeRha and the template DtRha.


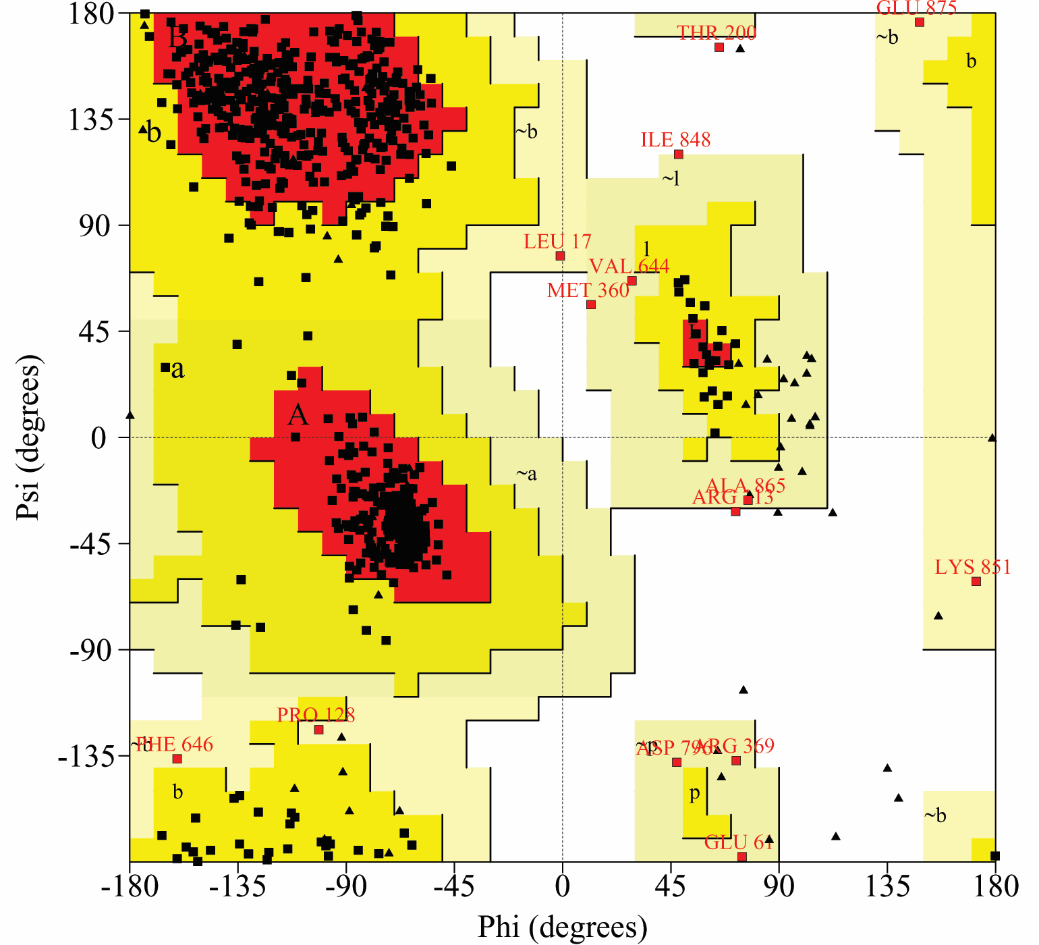


**Fig. S2.** The Ramachandran plot of TpeRha model.


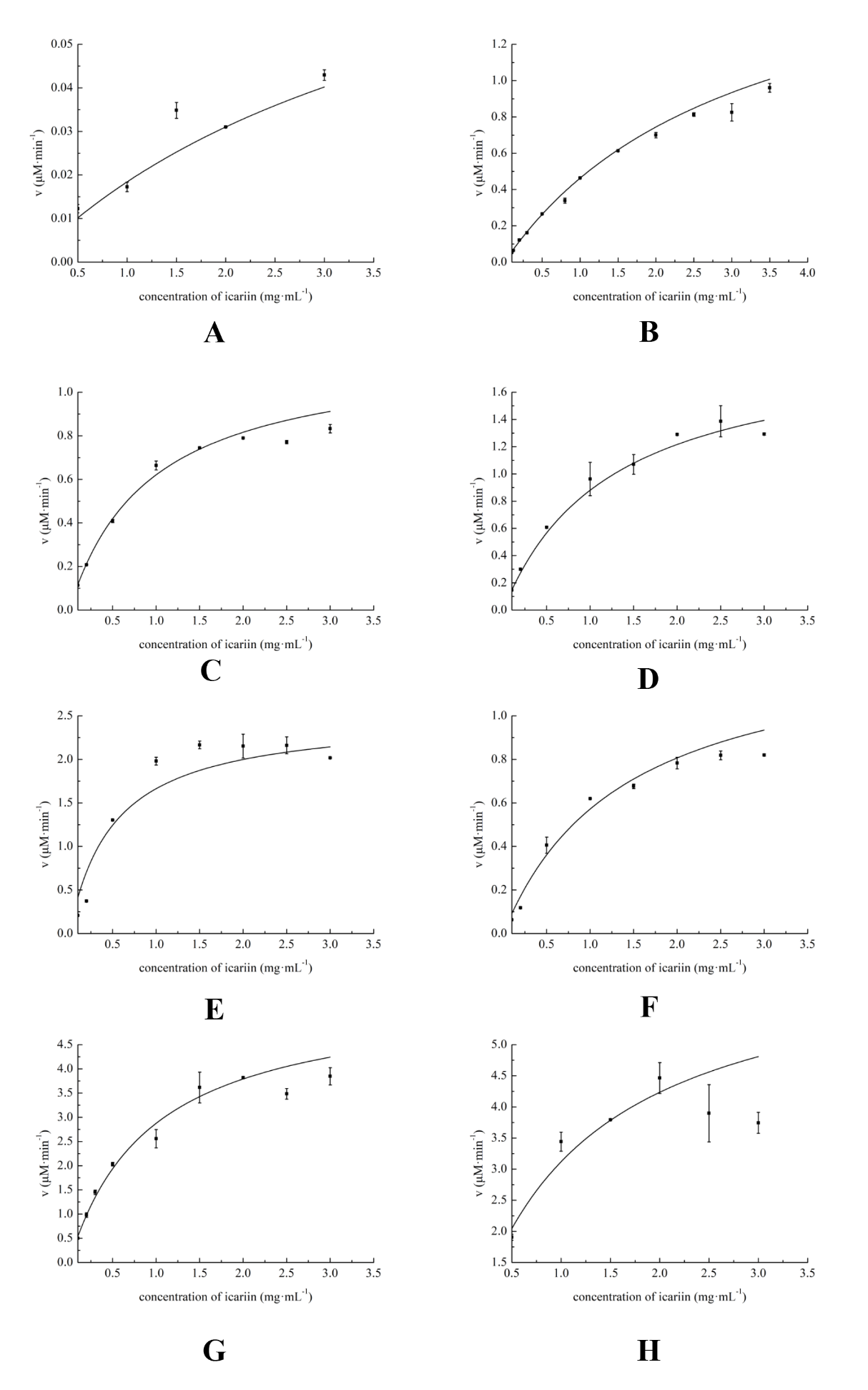


**Fig. S3.** The kinetic curves of TpeRha and mutants with different concentrations of icariin. (A) TpeRha, (B) D506A, (C) H570A, (D) K579A, (E) DH, (F) DK, (G) HK, (H) DHK.


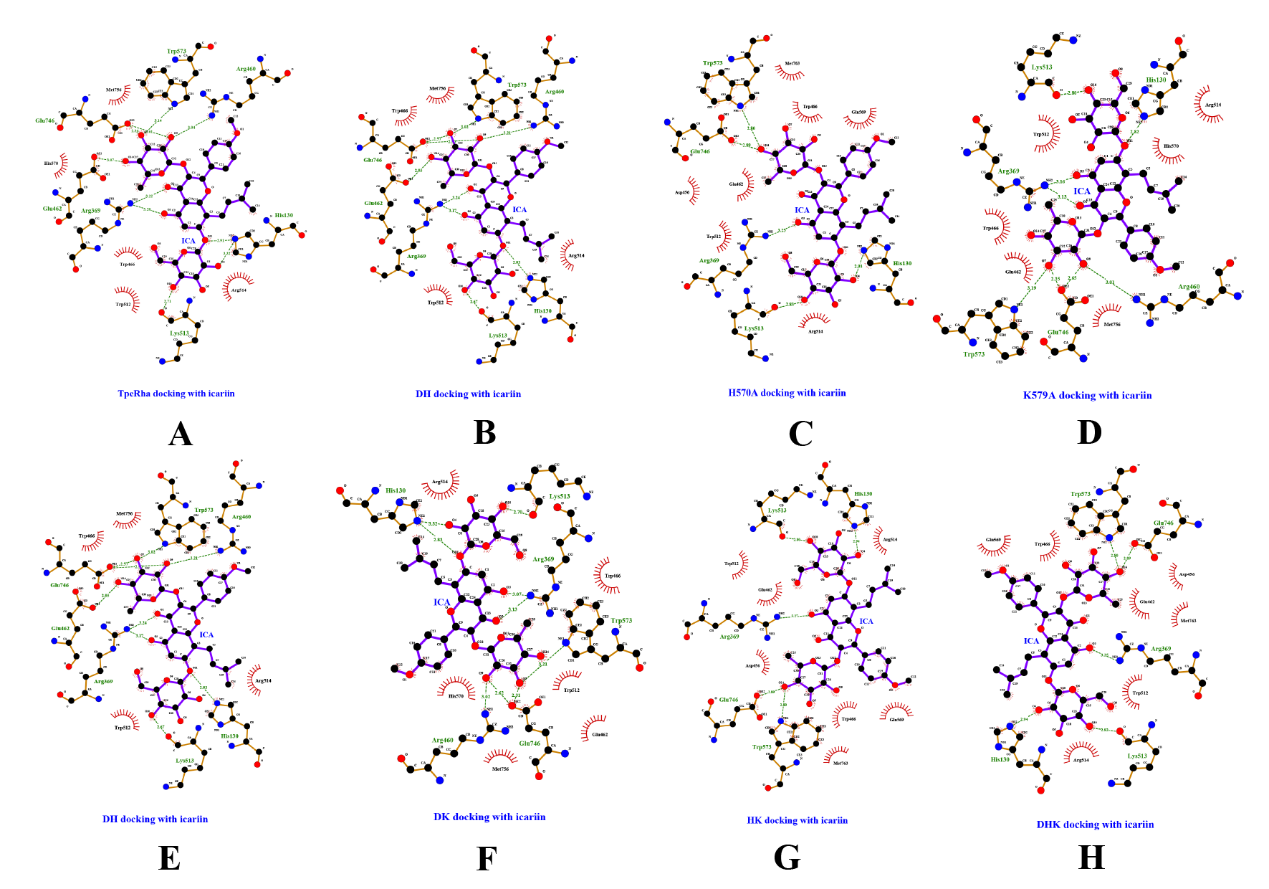


**Fig. S4.** The schematic results of receptor-ligand interaction between TpeRhas and icariin. (A) TpeRha, (B) D506A, (C) H570A, (D) K579A, (E) DH, (F) DK, (G) HK, (H) DHK. The hydrogen bonds between TpeRhas and icariin were shown as green dotted lines.


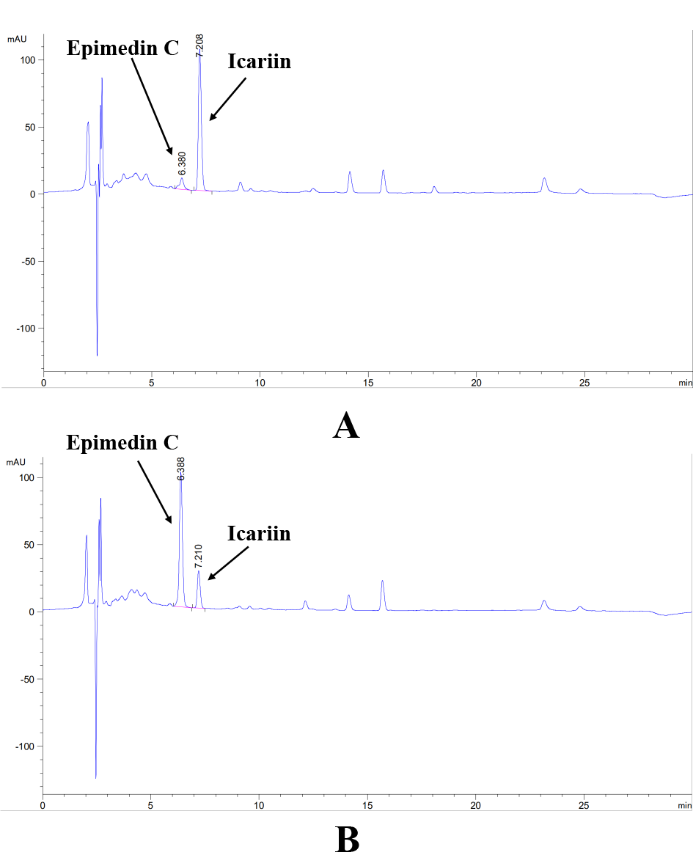


**Fig. S5.** The HPLC results of epimedin C hydrolyzed by TpeRha (A) and H570A (B).


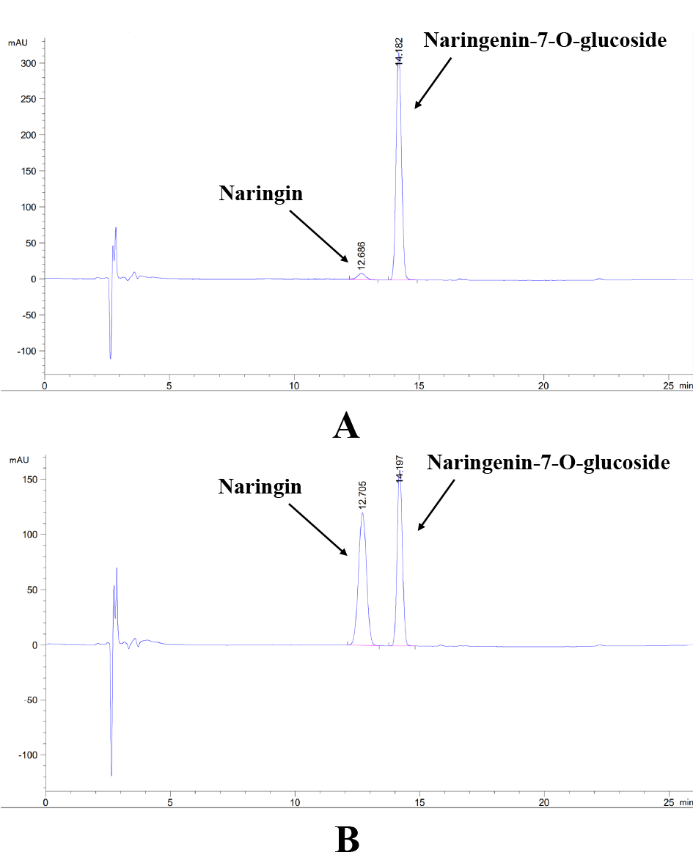


**Fig. S6.** The HPLC results of naringin hydrolyzed by TpeRha (A) and H570A (B).


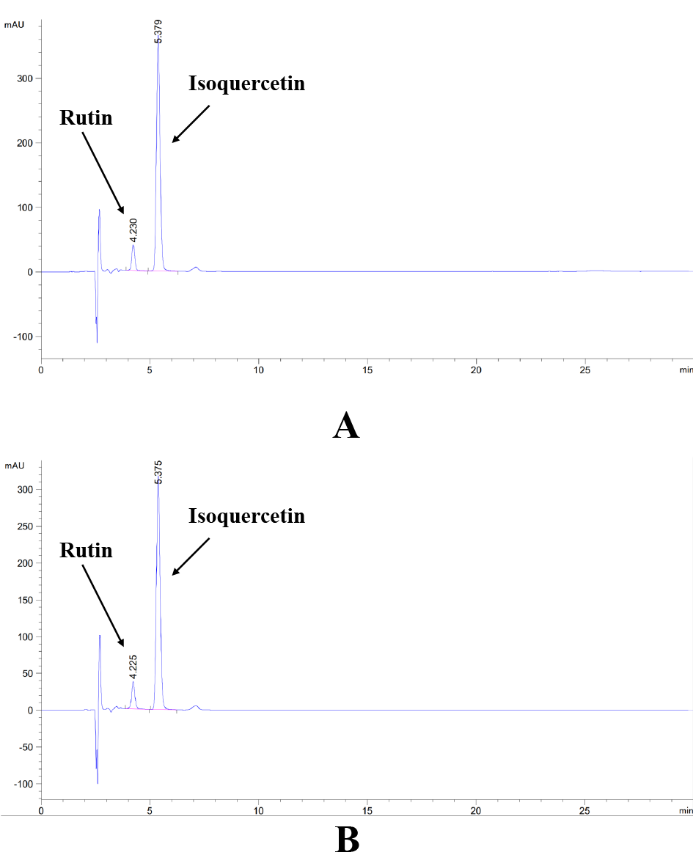


**Fig. S7.** The HPLC results of rutin hydrolyzed by TpeRha (A) and H570A (B).


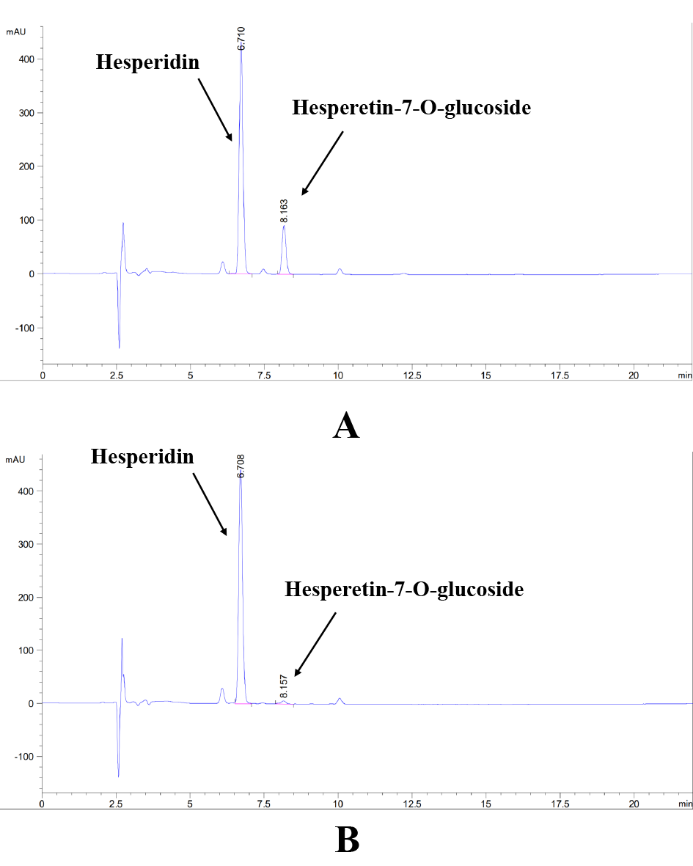


**Fig. S8.** The HPLC results of hesperidin hydrolyzed by TpeRha (A) and H570A (B).


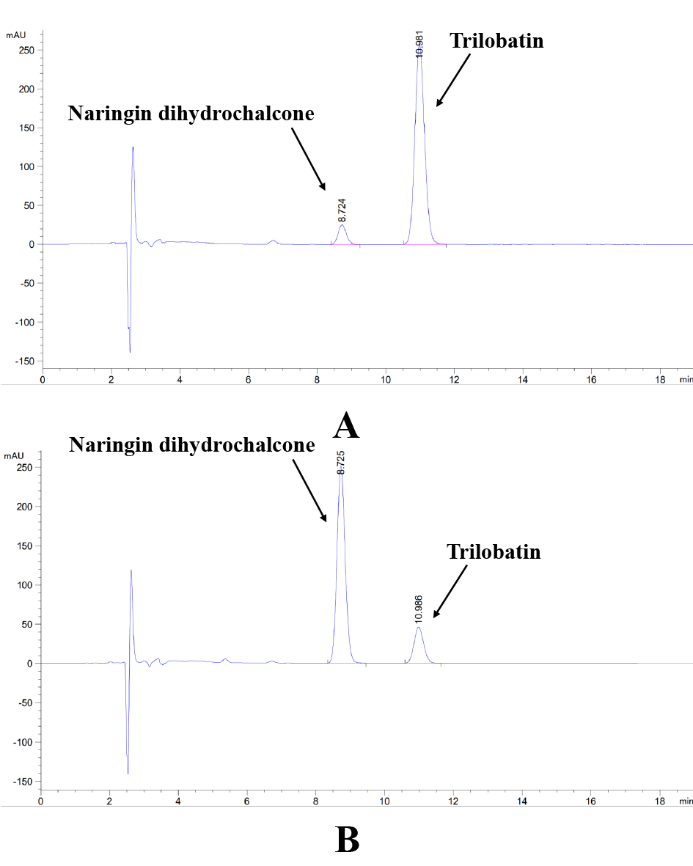


**Fig. S9.** The HPLC results of NDHC hydrolyzed by TpeRha (A) and H570A (B).


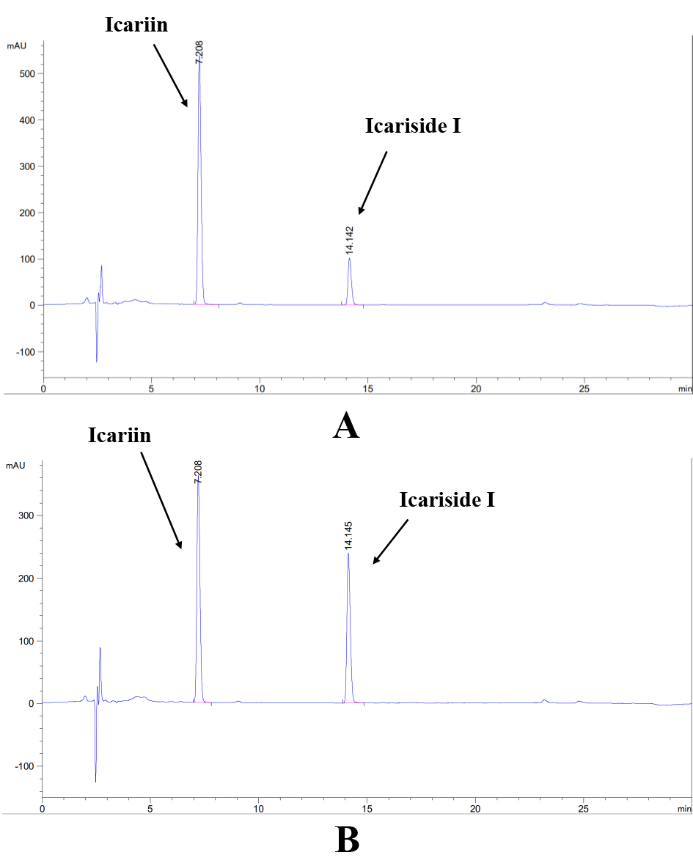


**Fig. S10.** The HPLC results of icariin hydrolyzed by TpeRha (A) and H570A (B).

**Table S1 The pairs of forward and reverse primers used for mutation.**

| **Primer name** | **Sequence (5’→3’, the corresponding mutant amino acid were labeled italic)** |
| --- | --- |
| E462AF | CGTGAT*GCA*CGTATGGGCTGGCTGGGTGAC |
| E462AR | CATACG*TGC*ATCACGCTGCGGGCAATCGGT |
| E746AF | CTGTGG*GCA*CGCTGGGAAAAACTGGAAGGC |
| E746AR | CCAGCG*TGC*CCACAGGGTGGTGGCACCGTT |
| H130AF | CCGGCC*GCA*CGTCATGAACTGTTTTATGCC |
| H130AR | ATGACG*TGC*GGCCGGATCTTTAAAGCTCAG |
| R369AF | AATCTG*GCA*CTGGCCCGCGCAACCGATGAA |
| R369AR | GGCCAG*TGC*CAGATTACGGGTATCCAGACG |
| R460AF | CCGCAG*GCA*GATGAACGTATGGGCTGGCTG |
| R460AR | TTCATC*TGC*CTGCGGGCAATCGGTCGGAAT |
| K513AF | TATTGG*GCA*CGCTATCCGAGCGATCCGGCC |
| K513AR | ATAGCG*TGC*CCAATACGGCGGTGCCACATC |
| W573AF | GGTGAC*GCA*TGTCCGCCGGGTGACAAATTT |
| W573AR | CGGACA*TGC*GTCACCATGCTGACCCAGTTT |
| D506AF | ATTAGT*GCA*GTGGCACCGCCGTATTGGAAA |
| D506AR | TGCCAC*TGC*ACTAATACTACCATCTTCTTT |
| H570AF | GGTCAG*GCA*GGTGACTGGTGTCCGCCGGGT |
| H570AR | GTCACC*TGC*CTGACCCAGTTTGGTCAGATG |
| K579AF | GGTGAC*GCA*TTTCCGAAACGTACCCCGCTG |
| K579A | CGGAAA*TGC*GTCACCCGGCGGACACCAGTC |
| H570YF | GGTCAG*TAT*GGTGACTGGTGTCCGCCGGGT |
| H570YR | GTCACC*ATA*CTGACCCAGTTTGGTCAGATG |
| D456AF | CCGACC*GCA*TGCCCGCAGCGTGATGAACGT |
| D456AR | CGGGCA*TGC*GGTCGGAATACCCATCAGATT |
| W466AF | ATGGGC*GCA*CTGGGTGACGCCCAGCTGACC |
| W466AR | ACCCAG*TGC*GCCCATACGTTCATCACGCTG |
| W521AF | CCGGCC*GCA*GGTACCGCCTATGCAACCATT |
| W521AR | GGTACC*TGC*GGCCGGATCGCTCGGATAGCG |
| M756AF | ACCGGT*GCA*AATAGCCATAATCATGTTATG |
| M756AR | GCTATT*TGC*ACCGGTGCCTTCCAGTTTTTC |
| H761AF | CATAAT*GCA*GTTATGCTGGGTAGCGTTGAT |
| H761AR | CATAAC*TGC*ATTATGGCTATTCATACCGGT |
| R747AF | TGGGAA*GCA*TGGGAAAAACTGGAAGGCACC |
| R747AR | TTCCCA*TGC*TTCCCACAGGGTGGTGGCACC |

**Table S2 The gradient elution conditions for epimedin C, icariin and icariside Ⅰ analysis**

| Time (min) | Acetonitrile (%) | 0.5% acetic acid (%) |
| --- | --- | --- |
| 0 | 30 | 70 |
| 10 | 50 | 50 |
| 20 | 85 | 15 |
| 25 | 30 | 70 |

**Table S3 The gradient elution conditions for naringin and naringenin-7-O-glucoside analysis.**

| Time (min) | Acetonitrile (%) | 0.5% acetic acid (%) |
| --- | --- | --- |
| 0 | 22 | 78 |
| 8 | 22 | 78 |
| 18 | 45 | 55 |
| 19 | 45 | 55 |
| 24 | 22 | 78 |
| 26 | 22 | 78 |

**Table S4 The gradient elution conditions for rutin and isoquercetin analysis.**

| Time (min) | Acetonitrile (%) | 0.5% acetic acid (%) |
| --- | --- | --- |
| 0 | 27 | 73 |
| 30 | 27 | 73 |

**Table S5 The gradient elution conditions for hesperidin and hesperetin-7-O-glucoside analysis.**

| Time (min) | Acetonitrile (%) | 0.5% acetic acid (%) |
| --- | --- | --- |
| 0 | 25 | 75 |
| 5 | 25 | 75 |
| 15 | 50 | 50 |
| 35 | 60 | 40 |
| 40 | 85 | 15 |
| 45 | 25 | 75 |

**Table S6 The gradient elution conditions for NDHC and trilobatin analysis.**

| Time (min) | Acetonitrile (%) | 0.5% acetic acid (%) |
| --- | --- | --- |
| 0 | 28 | 72 |
| 19 | 28 | 72 |
